# The Light-Fueled Self-Rotation of a Liquid Crystal Elastomer Fiber-Propelled Slider on a Circular Track

**DOI:** 10.3390/polym16162263

**Published:** 2024-08-09

**Authors:** Lu Wei, Yanan Chen, Junjie Hu, Xueao Hu, Yunlong Qiu, Kai Li

**Affiliations:** School of Civil Engineering, Anhui Jianzhu University, Hefei 230601, China; weilu@ahjzu.edu.cn (L.W.); cyn@stu.ahjzu.edu.cn (Y.C.); jjhu@stu.ahjzu.edu.cn (J.H.); hxa@stu.ahjzu.edu.cn (X.H.); ylqiu@stu.ahjzu.edu.cn (Y.Q.)

**Keywords:** self-rotation, liquid crystal elastomer, light fueled, slider, curved track

## Abstract

The self-excited oscillation system, owing to its capability of harvesting environmental energy, exhibits immense potential in diverse fields, such as micromachines, biomedicine, communications, and construction, with its adaptability, efficiency, and sustainability being highly regarded. Despite the current interest in track sliders in self-vibrating systems, LCE fiber-propelled track sliders face significant limitations in two-dime nsional movement, especially self-rotation, necessitating the development of more flexible and mobile designs. In this paper, we design a spatial slider system which ensures the self-rotation of the slider propelled by a light-fueled LCE fiber on a rigid circular track. A nonlinear dynamic model is introduced to analyze the system’s dynamic behaviors. The numerical simulations reveal a smooth transition from the static to self-rotating states, supported by ambient illumination. Quantitative analysis shows that increased light intensity, the contraction coefficient, and the elastic coefficient enhance the self-rotating frequency, while more damping decreases it. The track radius exhibits a non-monotonic effect. The initial tangential velocity has no impact. The reliable self-rotating performance under steady light suggests potential applications in periodic motion-demanding fields, especially in the construction industry where energy dissipation and utilization are of utmost urgency. Furthermore, this spatial slider system possesses the ability to rotate and self-vibrate, and it is capable of being adapted to other non-circular curved tracks, thereby highlighting its flexibility and multi-use capabilities.

## 1. Introduction

A self-oscillating system [1,2,3] refers to the phenomenon where a system relies on fixed environmental stimuli to trigger and induce continuous and stable periodic motion [4,5,6] without external drives. The system absorbs energy from external stimuli, inducing the periodic amplification of its energy. This results in periodic energy conversion within the system, compensating for the energy dissipation caused by damping in the process of motion. Through this positive feedback mechanism, the system is propelled to generate nonlinear responses [7] and amplify the control effect of the stimuli on its components or motion, enabling the system to reach a novel steady state, and consequently perform spontaneous periodic motion with invariant frequency [8,9,10]. The previous information implies that a self-rotating system does not require a sophisticated controller [11], which will result in more convenient and straightforward operation. Currently, the existing feedback mechanisms often involve multi-process coupling and internal adaptive feedback to achieve the purpose of energy compensation, such as the multi-process coupling of droplet evaporation with membrane deformation and movement [12], the coupling of air expansion with liquid column movement [13], the coupling mechanism in plate buckling and chemical reactions [14], the coupling of bridge vibrations with electrical energy [15], and photo-induced thermo-surface tension gradients [16,17]. Additionally, the period and amplitude in the state of self-rotating system equilibrium are dictated solely by the inherent properties of the system, regardless of the initial conditions. This unique characteristic guarantees the internal stability of the feedback system, enhancing its robustness [18,19,20] and facilitating the deeper comprehension of non-equilibrium thermodynamic theories. Given the unique characteristics and advantages of self-rotating systems, numerous self-rotating machines [21,22,23] have been created to explore their practical application potential. The commonly available self-rotating machines include miniaturized autonomous robots [24], nano-generators [25], energy harvesting and capture systems [26], active machines [27,28,29], autonomous separators [30], and mass transport equipment [31].

Moreover, the achievement of self-rotation necessitates the use of active materials capable of responding to external stimuli. Such materials include dielectric elastomers [32], liquid crystal elastomers, stimulation-responsive ionic gels [33], polyelectrolyte gels [34], shape memory polymers, smart polymer hydrogels [35], and thermotropic liquid crystalline polymer composites [36]. They possess immense application potential in diverse fields, including biomedicine [37], agriculture, transportation, corrosion prevention, and material science. LCEs, one of the emerging environmentally responsive materials [38,39,40], are polymeric networks formed by the crosslinking of liquid crystal monomers, possessing the characteristics of both liquid crystals and elastomers. Upon being exposed to external environmental stimuli, the liquid crystal units within LCEs transition from anisotropy to isotropy at the microscopic level, with the liquid crystal monomers undergoing rotation or phase changes [41]. This leads to the conversion of *trans-isomers* in the liquid crystal elastomer fibers to *cis-isomers*, causing the nonlinear unidirectional contraction of the LCEs macroscopically [42]. Once the stimulus is removed, the LCEs exhibit their inherent reversibility, enabling them to return to their original state, which contributes significantly to the realization of non-driven spontaneous periodic motion.

Different types of LCE fibers demonstrate distinct responses to a variety of external stimuli, such as light, heat, electricity, magnetism, and humidity. With continuous development and in-depth research on LCE fibers, other researchers have been able to fabricate LCEs that respond to a single or multiple coupled stimuli, including magnetically responsive LCEs [43,44], light-responsive LCEs [45,46], thermally responsive LCEs [47], humidity-responsive LCEs [48], electrically responsive LCEs [49,50], and multi-responsive LCEs [51,52]. The self-rotating modes based on the stimulus-responsive behavior of LCEs are constantly diversifying. They encompass various forms, such as vibration [53], bending [54,55], self-rotation [56], torsion [57,58], rolling [59], buckling [60], chaos [61,62], the sitting up of LCE thin film [44], eversion [63], and inversion, as well as the synchronized movement of multiple coupled self-oscillators [64].

Among the diverse stimulus-responsive LCEs, light emerges as a highly attractive clean energy source due to its ease of accessibility and controllability [65]. Meanwhile, light-fueled LCEs exhibit unique advantages [66,67,68], including significant strain resistance, reversible deformation, a rapid response, silence, etc. [69,70]. Therefore, the light–mechanical coupled self-rotating system stands out prominently in terms of both its application potential and value. The existing self-oscillating systems of LCEs have been applied in self-rotating engines [71], self-oscillating flexible circuits [72], self-fluttering aircraft [73], self-paddling boats [74], soft robots [75], self-moving automobiles [76], and other fields.

In the last few years, the intense focus on LCEs has been driven by extensive research into self-sliding systems [77,78,79], particularly with regard to track-mounted sliders [80,81]. However, the potential of these systems is still constrained by the limitations in in-plane motion, such as the difficulty in achieving self-rotation due to necessary deformation. To address this challenge, this paper proposes a spatial LCE fiber-propelled slider system that reduces the deformation of LCEs during energy conversion, enabling self-rotation. The main contents of this paper are outlined as follows: In Section 2, we establish a theoretical model of the system based on the proposed light–mechanical coupling dynamics and the deformation mechanism of light-responsive LCE fibers. In Section 3, we explore the system’s behavior in both the static and self-rotating states under constant illumination. Analysis reveals spontaneous periodic motion modes and the underlying mechanisms governing these modes. In Section 4, we quantitatively investigate the impact of critical dimensionless parameters on the cyclic frequency of the system through mathematical modeling and data calculations. Finally, we conclude the key findings of this paper.

## 2. Theoretical Model and Formulation

In this section, we first describe a newly designed light-fueled self-rotating dynamic system that comprises an LCE fiber, a slotted slider, and a rigid circular track. Subsequently, based on the dynamic mechanical model of the LCE optical fiber and the theorem of momentum moment, combined with spatial analytic geometry derivations, the dynamic control equation of the periodic self-rotating system is calculated. Finally, to address the dimensional impact among the parameters in the control equation, we normalize and standardize the parameters using the dimensionless method and introduce the process of numerical analytical calculation.

### 2.1. Dynamics of Self-Rotating System

**Figure 1:**
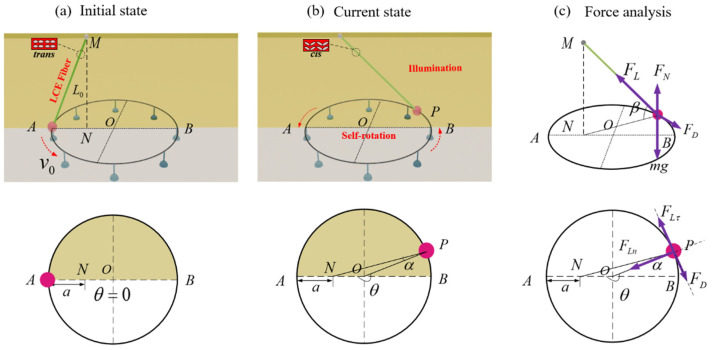
The side and top views of light-fueled self-rotating system with an LCE fiber, a slotted slider, and a rigid circular track: (**a**) initial state; (**b**) current state; and (**c**) force analysis. Under stable illumination, the slotted slider propelled by the LCE fiber can undergo spontaneous and continuous periodic motion on the circular track.

Figure 1 and Video S1 illustrate the spatial structural model of a light-fueled self-rotating system of an LCE fiber propelling a slider. This system consists of an LCE fiber, a slotted slider, and a rigid circular track with a radius of r. The LCE fiber has an original length of $L_{0}$, with one end attached to the horizontal fixed end and the other end connected to a slotted slider of mass m. The slider is firmly nested into the circular track through the slot, while the circular track itself is fastened stably to horizontal fixed supports. Under the given initial tangential velocity $v_{0}$ and the stimulation of the designed light, the slider propelled by the LCE fiber can perform self-sustaining periodic motion on the circular track. Given that the mass of the LCE fiber is significantly smaller than that of the slider of m, the influence of its mass on motion can be disregarded. In addition, while the slider is moving on the circular track, the slider is subjected to damping force, acting in the opposite direction of the slider’s motion. We took the center of the circular track as the origin o, with the vertical upward direction as the positive z axis, the horizontal rightward direction as the positive y axis, and the perpendicular outward direction in the paper as the positive x axis. We define point A as the intersection of the negative y axis and the circular track, which serves as the original position for movement. The connection point between the LCE fiber and the horizontal fixed end is labeled as point M, where the projection of M onto the xoy plane falls on the negative y axis, referred to as point N. Let P be the instantaneous position of the mass block, with the angles ∠NPO=α, ∠MPN=β, and ∠AOP=θ.

As shown in Figure 1a,b, the yellow area represents the illuminated zone with an angle $\theta_{0}$, ranging from π to 2π, while the colorless area represents the non-illuminated zone in this paper. Driven by the initial tangential velocity, the slider moves in a tangential direction within the plane, continuing its rotation until the slider reaches zero velocity within the illuminated area. At this moment, the *trans-isomers* in the LCE fibers transforms into the *cis-isomers* upon UV light irradiation, leading to the unidirectional contraction of the LCE fiber. This contraction propels the slider to continue moving in a counterclockwise direction, subsequently exiting the illuminated zone. Due to inertia, the slider continues to move in the non-illuminated zone at a decelerated speed, until it re-enters the illuminated zone and repeats the process. The repetition of this cycle results in spontaneous circular motion, termed self-rotation.

As depicted in Figure 1c, with the vertical forces acting on the slider cancelling each other out at all times, there is no chance of vertical displacement, allowing the slider to move solely within the plane of the circular track. Therefore, we only consider the dynamic response of the slider within the xoy plane. In Figure 1, θ is the angle which is the projection of the LCE string rotating angular displacement on the horizontal plane. When θ falls within the interval from 0 to π, based on the theorem of momentum moment, the mechanical control equation of the system is given as follows [82,83,84]:

(1) $$mr^{2}\ddot{\theta }=-{rF}_{L}\cos\beta \sin\alpha -{rF}_{D}$$

where $\ddot{\theta }$ refers to the angular acceleration of the slider at its instantaneous position, $F_{L}$ represents the tension of the LCE fiber, and $F_{D}$ denotes the damping force.

When θ lies between π and 2π, the equation is given as follows:

(2) $$mr^{2}\ddot{\theta }={rF}_{L}\cos\beta \sin\alpha -{rF}_{D}$$

The explanations of the variables $\ddot{\theta }$, $F_{L}$, and $F_{D}$ in Equation (2) are the same as those above in Equation (1). According to spatial geometric relationship of the structure in Figure 1c, we can determine that $\cos\beta =\frac{\sqrt{{\left(r-a\right)}^{2}+r^{2}-2r\left(r-a\right)\cos\theta }}{\sqrt{h^{2}+{\left(r-a\right)}^{2}+r^{2}-2r\left(r-a\right)\cos\theta }}$ and $\sin\alpha =\frac{\left(r-a\right)\sin\theta }{\sqrt{{\left(r-a\right)}^{2}+r^{2}-2r\left(r-a\right)\cos\theta }}$, where r is the radius of circular track, a represents the horizontal distance of the LCE fiber on the plane at the initial location, and h denotes the height of the LCE fiber in the z direction.

The tensile force in the LCE fiber is assumed to be directly proportional to the elastic strain and can be formulated as follows [85,86]:

(3) $$F_{L}=KL_{0}\varepsilon_{e}\left(t\right)$$

where K signifies the elastic coefficient in the LCE fiber, and $\varepsilon_{e}\left(t\right)$ designates the elastic strain present within the LCE fiber. To simplify analysis, the elastic strain $\varepsilon_{e}\left(t\right)$ in the case of small deformations can be approximated as a linear sum of the total strain $\varepsilon_{tot}\left(t\right)$ and the strain due to light-activated contraction $\varepsilon_{L}\left(t\right)$, namely, $\varepsilon_{tot}\left(t\right)=\varepsilon_{e}\left(t\right)+\varepsilon_{L}\left(t\right)$. Hence, the expression for the tension of the LCE fiber in Equation (3) can be rewritten as follows:

(4) $$F_{L}=KL_{0}{(\varepsilon }_{tot}\left(t\right)-\varepsilon_{L}\left(t\right))$$

For simplicity, we define the total strain $\varepsilon_{tot}\left(t\right)$ as the change in length from the original length $L_{0}$, expressed as $\varepsilon_{tot}\left(t\right)=\frac{L-L_{0}}{L_{0}}$. Consequently, the tension $F_{L}$ in Equation (4) can be reformulated as follows:

(5) $$F_{L}=K(L-L_{0}(1+\varepsilon_{L}\left(t\right)))$$

where L is the instantaneous length of the LCE fiber, which can be mathematically expressed as $L=\sqrt{h^{2}+{\left(r-a\right)}^{2}+r^{2}-2r\left(r-a\right)\cos\theta }$ using the cosine theorem of a triangle.

To simplify analysis, under the condition of low velocity, the damping force is typically modeled as a quadratic function, always acting in the opposite direction of the motion.

(6) $$F_{D}=\beta_{1}\dot{\theta }r+\beta_{2}{\left(\dot{\theta }r\right)}^{2}$$

where $\dot{\theta }$ denotes the angular velocity of the slider within a horizontal plane, $\beta_{1}$ represents the first damping coefficient, and $\beta_{2}$ signifies the second damping coefficient.

After integrating both Equations (5) and (6) into Equations (1) and (2), we can deduce the corresponding Equation (7) for the range of θ spanning from 0 to π and Equation (8) for the range between θ π and 2π.

(7) $$mr^{2}\ddot{\theta }=-Kr\left(1-\frac{L_{0}\left(1+\varepsilon_{L}\left(t\right)\right)}{\sqrt{h^{2}+{\left(r-a\right)}^{2}+r^{2}-2r\left(r-a\right)\cos\theta }}\right)\left(r-a\right)\sin\theta -\beta_{1}\dot{\theta }r^{2}-\beta_{2}{\left(\dot{\theta }\right)}^{2}r^{3}$$

(8) $$mr^{2}\ddot{\theta }=Kr\left(1-\frac{L_{0}\left(1+\varepsilon_{L}\left(t\right)\right)}{\sqrt{h^{2}+{\left(r-a\right)}^{2}+r^{2}-2r\left(r-a\right)\cos\left(2\pi -\theta \right)}}\right)\left(r-a\right)\sin\left(2\pi -\theta \right)-\beta_{1}\dot{\theta }r^{2}-\;\beta_{2}{\left(\dot{\theta }\right)}^{2}r^{3}$$

### 2.2. Dynamic Model of LCE

This section primarily focuses on describing the dynamic characteristics of the contraction strain induced by light in the LCE fibers. To simplify analysis, the light-induced contraction strain in the LCE fibers under small-scale deformation is considered to be directly correlated with the numerical fraction φ(t) of *cis-isomer* within the LCE fibers, i.e.,

(9) $$\varepsilon_{L}\left(t\right)=-C\varphi (t)$$

where C is the coefficient that characterizes the contraction of the LCE fiber.

Yu et al. [87] discovered that LCE fibers integrated with azobenzene moieties absorb UV light around 360 nm, enabling repeatable deformation without fatigue. Upon light exposure, molecular rearrangement leads to *trans–cis* isomerization and contraction. Azobenzene moieties convert light energy to a mechanical force, allowing for optical-to-mechanical coupling. When the LCE fiber is not illuminated, φ(t) remains zero, resulting in no contraction strain. However, upon light exposure, the *cis*-*isomer* φ(t) increases, triggering unidirectional contraction. This highlights that the *cis*-*isomer* fraction in the LCE fiber determines the degree of contraction strain under light stimulus.

Given the negligible effect of strain on the LCE’s *cis-trans* isomerization, we disregard it. The findings [88,89] shows that the fraction of *cis*-*isomers* is influenced by thermal excitation, thermally driven relaxation, and light-responsive isomerization. However, the thermal excitation’s impact is minor, so we omit it. Consequently, the governing equation for the *cis*-*isomer* fraction is simplified as follows:

(10) $$\frac{\partial \varphi \left(t\right)}{\partial t}=\eta_{0}I\left(1-\varphi \left(t\right)\right)-\frac{\varphi \left(t\right)}{T_{0}}$$

where $\eta_{0}$ denotes the light absorption constant, $T_{0}$ refers to the thermally driven relaxation time from the *cis* to the *trans* state, and I signifies the light intensity. By solving Equation (10), we can obtain the number fraction of the *cis-isomer*:

(11) $$\varphi \left(t\right)=\frac{\eta_{0}T_{0}I}{\eta_{0}T_{0}I+1}+\left(\varphi_{0}-\frac{\eta_{0}T_{0}I}{\eta_{0}T_{0}I+1}\right)exp\left[-\frac{t}{T_{0}}\left(\eta_{0}T_{0}I+1\right)\right]$$

where $\varphi_{0}$ represents the initial number fraction of *cis* photochromic molecules in the non-illuminated zone. For simplicity, we assume that $\varphi_{0}$ initially takes a value of zero upon entering the illuminated zone; thus Equation, (11) can be simplified as follows:

(12) $$\varphi \left(t\right)=\frac{\eta_{0}T_{0}I}{\eta_{0}T_{0}I+1}\left\{1-exp\left[-\frac{t}{T_{0}}\left(\eta_{0}T_{0}I+1\right)\right]\right\}$$

In the non-illuminated zone, by setting the value of I to zero, the *cis* number fraction of photosensitive molecules can be obtained as follows:

(13) $$\varphi \left(t\right)=\varphi_{0}exp\left(-\frac{t}{T_{0}}\right)$$

In Equation (12), at the initial time t=0, the maximum possible value of $\varphi_{0}$ is denoted as $\varphi_{0max}=\frac{\eta_{0}T_{0}I}{\eta_{0}T_{0}I+1}$. Substituting this value into Equation (12) yields the following:

(14) $$\varphi \left(t\right)=\frac{\eta_{0}T_{0}I}{\eta_{0}T_{0}I+1}exp\left(-\frac{t}{T_{0}}\right)$$

### 2.3. Nondimensionalization

It is evident that the numerical calculations in this study involve multiple parameters. To reveal the characteristic properties of the system and simplify the equations, the following dimensionless parameters are introduced: $\bar{\dot{\theta }}=\dot{\theta }T_{0},\;\bar{\ddot{\theta }}=\ddot{\theta }T_{0}^{2},\;\bar{t}=t/T_{0},\;\bar{K}=KT_{0}^{2}/m,\;\bar{I}=\eta_{0}T_{0}I_{0},\;\bar{\beta_{1}}=\beta_{1}T_{0}/m,\;\bar{\beta_{2}}=\beta_{2}r/m,\;\bar{\varphi }\left(t\right)=\varphi \left(t\right)\frac{\eta_{0}T_{0}I+1}{\eta_{0}T_{0}I}$. Substituting the dimensionless parameters into Equations (7) and (8), respectively, we can obtain Equations (15) and (16) in a dimensionless form:

(15) $$\bar{\ddot{\theta }}=-\bar{K}\left(1-\frac{L_{0}}{r}\frac{1+\varepsilon_{L}\left(t\right)}{\sqrt{1+\frac{h^{2}}{r^{2}}+{\left(1-\frac{a}{r}\right)}^{2}-2\left(1-\frac{a}{r}\right)\cos\theta }}\right)\left(1-\frac{a}{r}\right)\sin\theta -\bar{\beta_{1}}\bar{\dot{\theta }}-\bar{\beta_{2}}{\left(\bar{\dot{\theta }}\right)}^{2}$$

(16) $$\bar{\ddot{\theta }}=\bar{K}\left(1-\frac{L_{0}}{r}\frac{1+\varepsilon_{L}\left(t\right)}{\sqrt{1+\frac{h^{2}}{r^{2}}+{\left(1-\frac{a}{r}\right)}^{2}-2\left(1-\frac{a}{r}\right)\cos\left(2\pi -\theta \right)}}\right)\left(1-\frac{a}{r}\right)\sin\left(2\pi -\theta \right)-\bar{\beta_{1}}\bar{\dot{\theta }}-\bar{\beta_{2}}{\left(\bar{\dot{\theta }}\right)}^{2}$$

When entering the illuminated zone, Equation (12) can be simplified as follows:

(17) $$\bar{\varphi }\left(t\right)=1-exp\left[-\left(1+\bar{I}\right)\bar{t}\right]$$

When exiting the illuminated zone, Equation (14) can be simplified as follows:

(18) $$\bar{\varphi }\left(t\right)=exp\left(-\bar{t}\right)$$

Simultaneously, the horizontal tangential component $F_{L\tau }$ of the tension of the LCE fiber, as defined in Equation (5), and the damping force, as stated in Equation (6), can be expressed in a dimensionless form as follows:

When θ ranges from 0 to π, we can derive Equation (19):

(19) $$\bar{F_{L\tau }}=-\bar{K}\left(1-\frac{L_{0}}{r}\frac{1+\varepsilon_{L}\left(t\right)}{\sqrt{1+\frac{h^{2}}{r^{2}}+{\left(1-\frac{a}{r}\right)}^{2}-2\left(1-\frac{a}{r}\right)\cos\theta }}\right)\left(1-\frac{a}{r}\right)\sin\theta$$

When θ ranges from π to 2π, we can derive Equation (20):

(20) $$\bar{F_{L\tau }}=\bar{K}\left(1-\frac{L_{0}}{r}\frac{1+\varepsilon_{L}\left(t\right)}{\sqrt{1+\frac{h^{2}}{r^{2}}+{\left(1-\frac{a}{r}\right)}^{2}-2\left(1-\frac{a}{r}\right)\cos\left(2\pi -\theta \right)}}\right)\left(1-\frac{a}{r}\right)\sin\left(2\pi -\theta \right)$$

(21) $$\bar{F_{D}}=\bar{\beta_{1}}\bar{\dot{\theta }}+\bar{\beta_{2}}{\left(\bar{\dot{\theta }}\right)}^{2}$$

As observed from Equations (15) and (16), both the equations are second-order nonlinear differential equations, which makes it impossible to find precise solutions. Consequently, aiming for precision, we choose the fourth-order Runge–Kuttamethod to iteratively solve the nonlinear high-order ordinary differential equations, with MATLAB R2021a software facilitating numerical computations and analyses. By adjusting the relevant parameters in Equations (15) and (16), including the mean values of $\bar{I}$, $\bar{K}$, C, $\bar{\beta_{1}}$, and $\bar{\beta_{2}}$, we can attain the self-rotation of the system. At the same time, we can obtain the tensile force, the damping force, the contraction strain, the angular velocity, and the position of the LCE fiber light–mechanical coupling system under instantaneous conditions.

## 3. Two Dynamic States and Mechanism of Self-Rotation

In this section, utilizing the control equations outlined in Section 2, we analyze the dynamic response of the light-fueled self-rotating system when it is subjected to constant illumination. Initially, we present two characteristic dynamic modes of the static state and the self-rotating state. Following this, we describe the underlying mechanisms that enable self-rotation.

### 3.1. Two Dynamic States

Before investigating the self-rotating dynamic behavior and photoresponsive characteristics of the system, it is necessary to obtain the range of actual typical values for the dimensionless parameters. Based on the existing experimental verifications and research results [90,91], the specific property parameter values of the materials and structure are presented in Table 1. The corresponding dimensionless parameter values required in this study are shown in Table 2.

**Figure 2:**
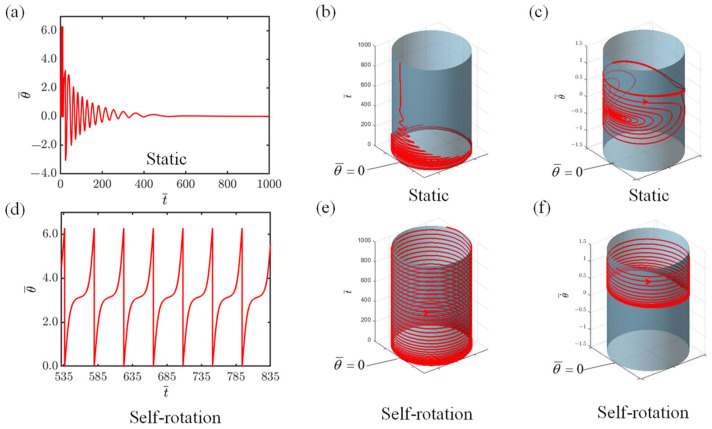
The two characteristic dynamic states of the system during constant exposure to light: the static state and the self-rotating state. (**a**,**b**) Time–history graph of angular displacement when $\bar{I}=0.2$. (**c**) Phase trajectory plot when $\bar{I}=0.2$. (**d**,**e**) Time–history graph of angular displacement when $\bar{I}=0.8$. (**f**) Phase trajectory plot when $\bar{I}=0.8$.

The time–history graph and phase trajectory plot for the system are attainable through the numerical solution of Equations (15) and (16), presented in Figure 2. The findings reveal the existence of two characteristic dynamic states of the system, namely, the static state and the self-rotating state, during constant exposure to light of $\bar{I}=0.2$ and $\bar{I}=0.8$. During the numerical simulation, we establish the following dimensionless variables for the system: C=0.3, $\bar{K}=1.0$, $\bar{v_{0}}=1.3$, $\bar{\beta_{1}}=0.015$, $\bar{\beta_{2}}=0.005$, $\bar{r}=1.5$, $\bar{L_{0}}=5$, $\bar{a}=0.5$,$\;\theta_{0}=\pi \sim 2\pi$. When $\bar{I}=0.2$, initially, the mass block rotates counterclockwise for two revolutions. Subsequently, it begins to rotate clockwise and counterclockwise in an alternating manner, with the rotating angle and angular velocity gradually decreasing, and ultimately settling at zero as a result of the damping force, indicating that it has reached a static state. Time–history curves of the vibrational response during this process are depicted in Figure 2a,b. The corresponding phase trajectory plot in Figure 2c shows that the motion trajectory eventually stabilizes at a single point. When $\bar{I}=0.8$, the angular velocity of the slider gradually stabilizes, indicating that the system has entered a self-rotating state, as shown in Figure 2d,e. Eventually, the maintenance of a limit cycle, resembling the phase trajectory in Figure 2f, exemplifies a periodically stable operational mode.

### 3.2. Mechanism of Self-Rotation

**Figure 3:**
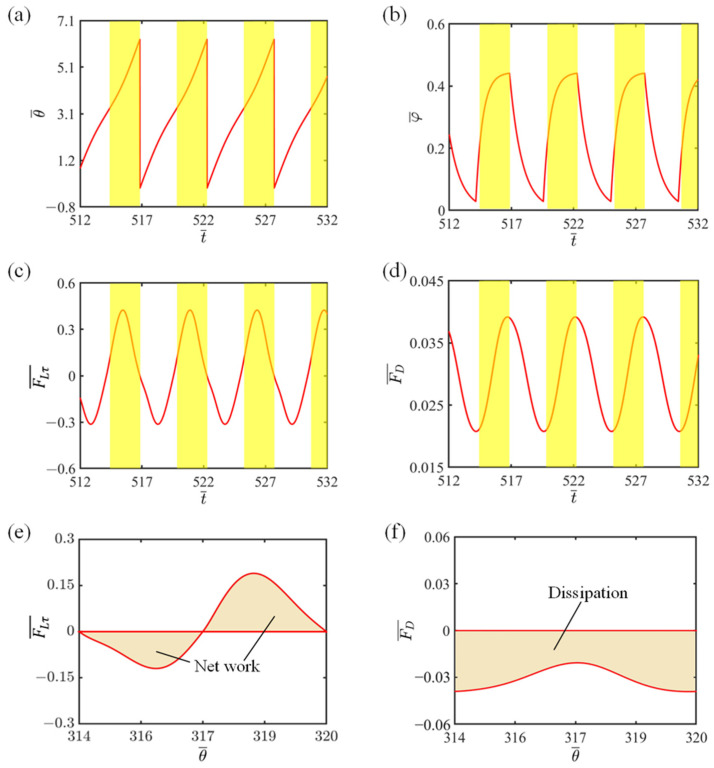
The self-rotating mechanism of the system. (**a**) The variation in rotating angle with time. (**b**) The variation in the number fraction of *cis*-*isomers* in the LCE fiber with time. (**c**) A time–history curve of horizontal tangential tension of LCE fiber. (**d**) A time–history curve of damping force. (**e**) Rotating angle-dependent horizontal tangential tension in the LCE fiber. (**f**) The rotating angle-dependent damping force.

In the investigation of the self-rotating mechanism, we particularly focus on how the system counters energy loss stemming from the damping forces. To further clarify this intricate dynamic, we utilize the visual aid of relationship curves to highlight the intricate connections between the critical variables that contribute to the self-rotating process, as visualized in Figure 3. For the purpose of analysis, we choose the following dimensionless variables of $\bar{I}=0.8$, C=0.3, $\bar{K}=1.0$, $\bar{v_{0}}=1.3$, $\bar{\beta_{1}}=0.015$, $\bar{\beta_{2}}=0.005$, $\bar{r}=1.5$, $\bar{L_{0}}=5$, $\bar{a}=0.5$, with $\theta_{0}$ ranging from π to 2π. In Figure 3a, we observe the changes in the rotating angle of the system as time progresses. The illuminated region, highlighted in yellow, indicates where the LCE fiber absorbs light. It is noticeable that the self-rotating system exhibits a consistent pattern, with the slider rotating repeatedly between the illuminated and non-illuminated sections. In Figure 3b, the fluctuation of the LCE fiber’s number fraction over time is revealed in relation to light exposure. When the rotating angle of the mass exceeds π, the LCE fiber comes into the illuminated areas, triggering a gradual rise in its number fraction towards a defined maximum. However, as the slider shifts from the illuminated to the non-illuminated regions, the LCE fiber’s number fraction drops sharply to zero. This recurring pattern of the system’s traversal between the illuminated and non-illuminated zones results in the periodic variations observed in the LCE fiber’s number fraction.

Figure 3c demonstrates the temporal evolution of tension in the LCE fiber. The cyclical self-rotating motion of the system is responsible for the periodic changes in tension. As the LCE fiber moves into the illuminated areas, the increased number fraction of the LCE fiber leads to a corresponding rise in contraction strain, accompanied by an augmentation in elastic strain. This ultimately results in an increment in tension within the LCE fiber. Conversely, when the system exits the illuminated regions, the tension decreases due to the reversal of light-induced contraction. As illustrated in Figure 3c, the variation in horizontal tangential tension of the LCE fiber is consistent with the theoretical framework presented in this study, particularly Equation (5). Figure 3d shows a time–history curve of the damping force, which also follows a period cycle. In the non-illuminated region, the damping force decreases, while in the illuminated region, it increases. This is due to the fact that the damping force is directly proportional to velocity, and as depicted in Figure 2f, the velocity initially decreases before subsequently increasing over time.

To gain a deeper understanding of the system’s energy absorption and compensation mechanism, we chart the dependency of horizontal tangential tension on a rotating angle, as depicted in Figure 3e, and we also represent the relationship of damping force with the rotating angle in Figure 3f. The hysteresis loop in Figure 3e shows LCE fiber’s net work (0.913) in a rotating cycle, balancing the absorbed energy from light-responsive contraction and released energy during recovery. The loop in Figure 3f quantifies the damping force’s energy consumption (also 0.913). These two balance, indicating the damping force losses are precisely compensated by the LCE fiber’s energy differences. This demonstrates that the LCE fiber-propelled slider system maintains its periodic rotation effectively.

## 4. Parameter Study

In the previous section, we analyze the dynamic behavior of the slider propelled by the light-fueled LCE fiber based on Equations (15)–(21) and the following dimensionless physical parameters: $\bar{I}$, C, $\bar{K}$, $\bar{v_{0}}$, $\bar{\beta_{1}}$, $\bar{\beta_{2}}$, $\bar{r}$, $\bar{L_{0}}$, $\bar{a}$, and $\theta_{0}.$ In this section, under the condition that $\bar{L_{0}}=5$, $\bar{a}=0.5$,$\;and\;\bar{\beta_{2}}=0.005$, and with the illumination region remaining stable within the range $\theta_{0}$ of π to 2π, we proceed to conduct quantitative analysis on the dynamic impact of each of the six major dimensionless parameters, i.e., $\bar{I}$, C, $\bar{K}$, $\bar{v_{0}}$, $\bar{\beta_{1}}$, and $\bar{r}$, specifically focusing on how they affect the self-rotating frequency, denoted as f.

### 4.1. Effect of Light Intensity

**Figure 4:**
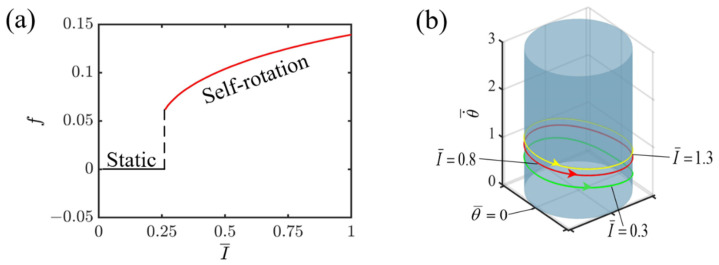
Effect of light intensity on self-rotating frequency. (**a**) Frequency variations with light intensities. (**b**) Depictions of limit cycles at $\bar{I}=0.3$, 0.8, and 1.3.

Given the specified dimensionless variables, C=0.3, $\bar{K}=1.0$, $\bar{v_{0}}=1.3$, $\bar{\beta_{1}}=0.015$, and $\bar{r}=1.5$, Figure 4 illustrates how the intensity of light affects the self-rotating mechanism of the slider propelled by the light-fueled LCE fiber. As shown in Figure 4a, there is direct proportionality between the light intensity and its impact on frequency, indicating that as the intensity of light rises, the frequency also increases. This is due to the fact that higher light intensities empower the LCE fiber to absorb a larger quantity of energy and convert it into kinetic energy, which enables the system to cycle through a full revolution more quickly. As evident from Figure 4a, the key intensity of light that divides the static state and the self-rotating state is $\bar{I}=0.25$. Below this intensity of 0.25, the LCE fiber fails to absorb enough light energy to counter damping dissipation, leading to the transition into a static state due to its inability to maintain motion. Conversely, when the light intensity surpasses 0.25, the LCE fiber absorbs sufficient energy to overcome damping dissipation, enabling it to sustain a continuous and stable self-rotation, which defines the self-rotating state. In Figure 4b, the respective limit cycles for self-rotation are exhibited for various $\bar{I}$ values, including 0.3, 0.8, and 1.3. It is evident that as the light intensity rises at any given point on the circular ring, the velocity of the slider’s rotation increases significantly. This observation strongly suggests that boosting the light intensity plays a pivotal role in improving the energy utilization efficiency of the LCE fiber-propelled slider system.

### 4.2. Effect of Contraction Coefficient of LCE

**Figure 5:**
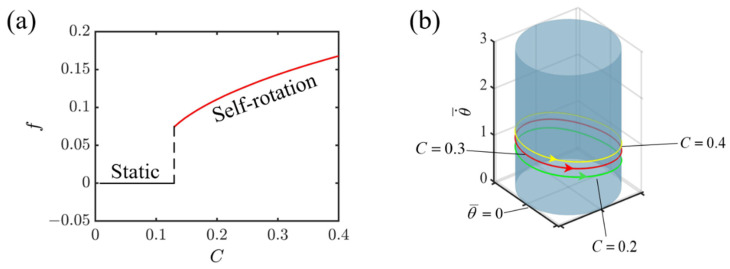
Effect of contraction coefficient on self-rotating frequency. (**a**) Frequency variations with contraction coefficient. (**b**) Depictions of limit cycles at C=0.2, 0.3, and 0.4.

Given the specified dimensionless variables, $\bar{I}=0.8$, $\bar{K}=1.0$, $\bar{v_{0}}=1.3$, $\bar{\beta_{1}}=0.015$, $\bar{r}=1.5$, Figure 5 illustrates how the contraction coefficient of the LCE affects the self-rotating mechanism of the slider propelled by the light-fueled LCE fiber. As depicted in Figure 5a, there is a clear limiting value for the contraction coefficient, mathematically identified as 0.12, marking a critical point for initiating self-rotation. Below this value of 0.12, the slider remains stationary. Nonetheless, upon exceeding 0.12, the system transitions into a state of self-rotation. Moreover, there is a tendency for the frequency to rise as C increases, which stems from the decrease in the LCE fiber’s capacity to absorb light, triggered by a reduction in the contraction coefficient, ultimately causing a decrease in the kinetic energy and frequency of the system. In Figure 5b, for various C values, including 0.2, 0.3, and 0.4, the corresponding limit cycles for self-rotation are displayed. In addition, at any fixed position on the circular ring, the increase in the contraction coefficient is accompanied by a marked augmentation in the slider’s rotational velocity. The observation indicates that augmenting the contraction coefficient of an LCE fiber can enhance the efficient transformation of light energy into mechanical energy.

### 4.3. Effect of Elastic Coefficient of LCE

**Figure 6:**
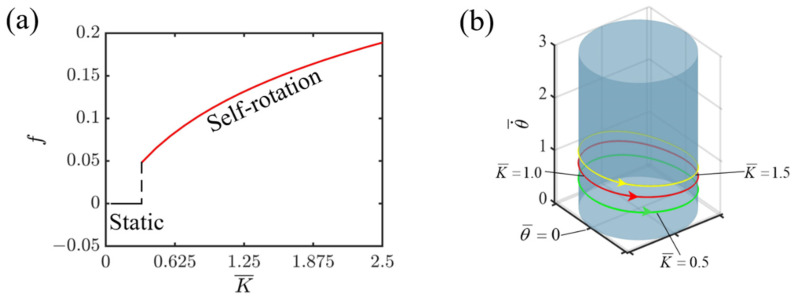
Effect of elastic coefficient on self-rotating frequency. (**a**) Frequency variations with elastic coefficient. (**b**) Depictions of limit cycles at $\bar{K}=0.5$, 1.0, and 1.5.

Given the specified dimensionless variables, $\bar{I}=0.8$, C=0.3, $\bar{v_{0}}=1.3$, $\bar{\beta_{1}}=0.015$, $\bar{r}=1.5$, Figure 6 illustrates how the elastic coefficient of the LCE affects the self-rotating mechanism of the slider propelled by the light-fueled LCE fiber. As illustrated in Figure 6a, the elastic coefficient serves as a crucial factor in determining the frequency of self-rotation. As the elastic coefficient rises, so does the frequency of self-rotation. This is attributed to the fact that a higher elastic coefficient yields a stronger elastic force from the LCE fiber. Consequently, the system accumulates more elastic potential energy, which is then converted into kinetic energy, ultimately resulting in a greater frequency of self-rotation. As seen in Figure 6a, an elastic coefficient of 0.42 acts as the vital value between the static and self-rotating modes for the system. Under continuous illumination, if the elastic coefficient falls below 0.42, the LCE fiber cannot harvest enough light energy to overcome the damping force, resulting in a static mode. Conversely, when the coefficient exceeds 0.42, the LCE fiber accumulates sufficient energy to counter the damping force and maintain continuous self-rotation. Figure 6b shows the respective limit cycles of self-rotation corresponding to the elastic coefficients of $\bar{K}=0.5$, 1.0, and 1.5. Notably, when considering a specific point on the circular track, an increase in the elastic coefficient $\bar{K}$ leads to a corresponding acceleration in the slider’s velocity, thereby enhancing the frequency. Therefore, when designing an LCE propelling system, selecting the appropriate elastic coefficient is crucial to achieving a superior performance.

### 4.4. Effect of Initial Tangential Velocity

**Figure 7:**
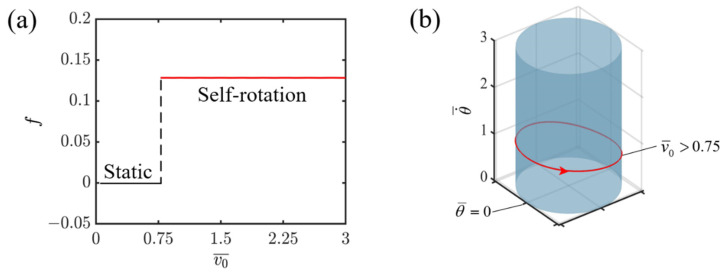
Effect of initial tangential velocity on self-rotating frequency. (**a**) Frequency variations with initial tangential velocity. (**b**) Depictions of limit cycles at $\bar{v_{0}}=1.1$, 1.3, and 1.5.

Given the specified dimensionless variables, $\bar{I}=0.8$, C=0.3, $\bar{K}=1.0$, $\bar{\beta_{1}}=0.015$, $\bar{r}=1.5$, Figure 7 illustrates how the initial tangential velocity affects the self-rotating mechanism of the slider propelled by the light-fueled LCE fiber.

Figure 7a depicts the relationship between the frequency of self-rotation and the initial tangential velocity. It is clearly shown that under constant illumination, the initial tangential velocity does not affect the system’s frequency. This is because the frequency of self-rotation is primarily determined by the interaction between the energy dissipated by the damping force and the net work generated by the light-fueled LCEs. These internal dynamics, together with the material properties, constitute the inherent characteristics of the system. It can be seen that when the initial tangential velocity is less than 0.75, the system attains a static state. This is attributed to the fact that with such a low initial tangential velocity, the LCE fiber fails to enter the illumination zone, thus preventing it from capturing sufficient light energy to sustain its dynamic movement. Conversely, when the initial tangential velocity surpasses 0.75, specifically at $\bar{v_{0}}=1.1$, 1.3, and 1.5, the system transitions into a self-rotating state. Furthermore, the corresponding limit cycle remains the same for $\bar{v_{0}}=1.1$, 1.3, and 1.5, as depicted in Figure 7b. The results show that when designing an LCE propelling self-rotating system, the initial velocity has little impact on the system performance as long as it can trigger self-rotation.

### 4.5. Effect of the First Damping Coefficient

**Figure 8:**
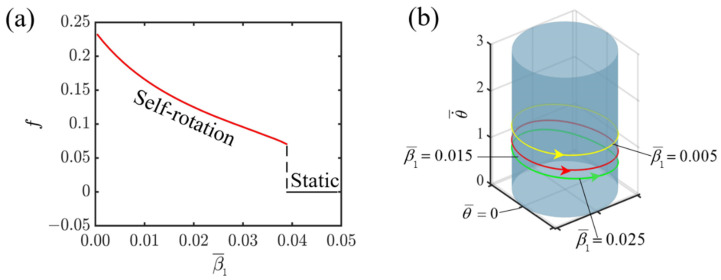
Effect of the first damping coefficient on self-rotating frequency. (**a**) Frequency variations with the first damping coefficient. (**b**) Depictions of limit cycles at $\bar{\beta_{1}}=0.005$, 0.015, and 0.025.

Given the specified dimensionless variables, $\bar{I}=0.8$, C=0.3, $\bar{K}=1.0$, $\bar{v_{0}}=1.3$, $\bar{r}=1.5$, Figure 8 illustrates how the first damping coefficient affects the self-rotating mechanism of the slider propelled by the light-fueled LCE fiber. As can be observed from Figure 8a, with the increase in the first damping coefficient, the system frequency gradually decreases. When the damping coefficient exceeds the critical value of 0.04, the system changes from a self-rotating state to a static state. The reason for this is that as the damping coefficient increases, the dissipative energy generated by the damping force also increases. When the slider propelled by the light-fueled LCE fiber enters the illuminated area, the energy collected becomes insufficient to overcome the increased dissipative energy, ultimately leading the system to enter a static state. When the system is in a self-rotating state, numerical calculations are performed with different values of $\bar{\beta_{1}}$, specifically 0.005, 0.015, and 0.025. The results indicate that as the first damping coefficient increases, the corresponding limit cycle shifts downwards in the depiction. Conversely, for smaller damping coefficients, the limit cycle is positioned higher, as illustrated in Figure 8b. Consequently, decreasing the damping coefficient of the medium facilitates the efficient transformation of light energy into mechanical energy.

### 4.6. Effect of Radius of Circular Track

**Figure 9:**
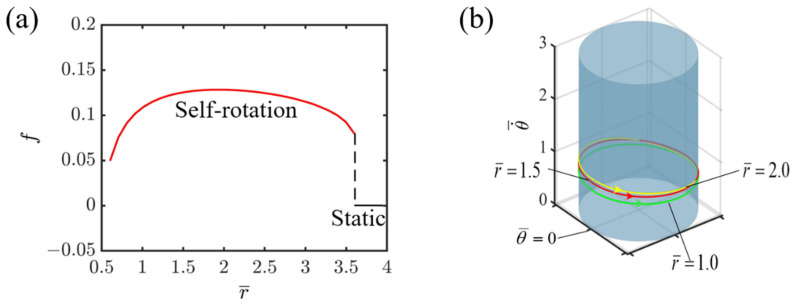
Effect of radius of circular track on self-rotating frequency. (**a**) Frequency variations with radius of circular track. (**b**) Depictions of limit cycles at $\bar{r}=1.0$, 1.5, and 2.0.

Given the specified dimensionless variables, $\bar{I}=0.8$, C=0.3, $\bar{K}=1.0$, $\bar{v_{0}}=1.3$, $\bar{\beta_{1}}=0.015$, Figure 9 illustrates how the radius of circular track affects the self-rotating mechanism of the slider propelled by the light-fueled LCE fiber. As shown in Figure 9a, as the radius increases, the system’s rotational frequency also increases. Owing to the increase in radius, there is more conversion of light energy from the light-fueled LCE into mechanical energy, which leads to an increase in the system’s internal kinetic energy. In the self-rotating state, a larger radius enables the system to complete more rotation cycles per unit time, thus increasing the rotational frequency. However, when the radius exceeds the threshold value of 2.0, the situation changes. At this point, as the radius continues to increase, the system’s rotational frequency begins to decrease until it finally enters a static state. The reason for this is that as the radius increases, the damping force experienced by the system also increases, and these damping forces consume the energy of the system’s rotation. When the dissipated energy reaches a certain level, the system may no longer be able to maintain its self-rotating state, and eventually enters a static state. Furthermore, as the radius continues to increase, it becomes increasingly challenging for the slider propelled by the light-fueled LCE fiber to enter the illuminated area. Consequently, the LCE fiber is unable to gather sufficient light energy to overcome the damping-induced energy losses, which ultimately leads to the gradual transition into a stationary state. It can also be observed from Figure 9a that the radius starts at 0.5 due to the assumption that the horizontal projected distance $\bar{a}$ of the LCE string is less than the radius. Figure 9b presents the corresponding limit cycles of self-rotation for $\bar{r}=1.0$,$\;\bar{r}=1.5$, and $\bar{r}=2.0$. It is evident that in the self-rotating state, the limit cycle with a larger radius experiences faster velocity variation. This is due to the negative work completed by the tensile force of the LCE before entering the illuminated region. The larger the radius is, the longer the elastic LCE becomes, coupled with the dissipative capability caused by damping, resulting in a more significant impact on the velocity. The findings suggest that the selection of the radius of circular track is essential for enhancing the energy efficiency of the LCE fiber-propelled slider system.

## 5. Conclusions

Despite the current interest in track sliders incorporating LCE fibers and their adaptability, efficiency, and sustainability in self-vibrating systems, their movement within a two-dimensional space is severely restricted. Specifically, self-rotation, a process requiring the intricate shaping deformation of the sliders and the LCE fiber-based system, poses a significant challenge. This limitation highlights the need for developing more flexible and mobile track slider designs with enhanced kinematic capabilities. To address these challenges, we present a novel light-fueled spatial system comprising an LCE fiber, a slotted slider, and a rigid track. This innovative design ensures the smooth self-rotation of the slider on a circular track under constant illumination, overcoming deformation issues during operation. Based on the dynamic mechanical model of the LCE optical fiber and the theory of momentum moment, combined with spatial analytic geometry derivations, we have derived the dimensionless dynamic control equation for the periodic self-rotating system. Utilizing the established fourth-order Runge–Kutta method and MATLAB R2021a software, we numerically solved the dynamic control equations. Our findings reveal two distinct motion states of the self-rotating slider system: the static state and the self-sliding state. Notably, we elaborate on the self-rotating process and its accompanying energy balancing mechanism. Here, the consistent external energy source compensates for the dissipation caused by system damping, thereby maintaining the dynamic equilibrium of the system.

In addition, quantitative analysis was carried out on the light intensity, the contraction coefficient, the elastic coefficient, the initial tangential velocity, the damping coefficient, and the radius of the circular track. The numerical calculation results show that the increase in the light intensity, the contraction coefficient, and the elastic coefficient lead to an increase in the self-rotating frequency. In contrast, the increase in the damping coefficient results in a significant decrease in the self-rotating frequency. Compared with the monotonic influence of the other parameters, the effect of the track radius on the frequency is non-monotonic. As the radius increases, the frequency first increases, and then decreases, and finally tends to be static. It is worth noting that the initial tangential velocity has no effect on the frequency of the system.

Although the simplicity, flexibility, and diverse motion capabilities of the proposed LCE fiber-propelled slider system promise widespread adoption, limitations persist. Notably, the small-scale deformation assumption oversimplifies the mechanical behavior, and the exclusion of viscoelastic effects in the LCE fibers, crucial under large actuation strains, introduces challenges. Viscoelasticity’s time-dependent nature causes hysteresis and energy dissipation, reducing energy efficiency by hindering mechanical work extraction from photomechanical coupling. Delayed deformation recovery further threatens motion stability in dynamic environments with fluctuating illumination. To enhance the system’s full potential, future research will integrate viscoelasticity into the model, enabling a deeper understanding of its impact on energy efficiency and stability. Additionally, exploring the system’s dynamic behavior under variable illumination and non-circular trajectories will enhance its robustness and adaptability in complex environments.

## Figures and Tables

**Table 1:** Material properties and geometric parameters.

Parameter	Definition	Value	Unit
I	light intensity	0–80	kW/m^2^
C	contraction coefficient of LCE fiber	0–0.4	/
K	elastic coefficient of LCE fiber	20–40	N/m
$T_{0}$	*Cis* to *trans* thermal relaxation time	0.02–0.45	s
$\eta_{0}$	light absorption constant	0.002	m^2^/(s·W)
m	mass of the slider	0–0.02	kg
$\beta_{1}$	the first damping coefficient	0–0.3	kg/s
$\beta_{2}$	the second damping coefficient	0–0.15	kg/m
$v_{0}$	initial tangential velocity	0–5	m/s
$\theta_{0}$	range of illuminated zone	0–2π	rad
r	radius of circular track	0.01–5	m
a	horizontal projection distance of LCE string	0.01–2.5	m
$L_{0}$	original length of LCE fiber	0.1–5	m

**Table 2:** Dimensionless parameters.

Parameter	$\bar{I}$	C	$\bar{K}$	$\bar{v_{0}}$	$\theta_{0}$	$\bar{\beta_{1}}$	$\bar{\beta_{2}}$
Value	0–1	0–0.4	0–10	0–3	π–2π	0–0.2	0–0.1

## Data Availability

The original contributions presented in the study are included in the article/Supplementary Material, further inquiries can be directed to the corresponding author/s.

## Supplementary Materials

The following supporting information can be downloaded at: https://www.mdpi.com/article/10.3390/polym16162263/s1, Video S1: The process of self-rotation of the system.

## References

[1] Ding W. (2009). Self-Excited Vibration.

[2] Thomson W. (2018). Theory of Vibration with Applications.

[3] Jenkins A. (2013). Self-oscillation. Phys. Rep..

[4] Zhang Z., Duan N., Lin C., Hua H. (2020). Coupled dynamic analysis of a heavily-loaded propulsion shafting system with continuous bearing-shaft friction. Int. J. Mech. Sci..

[5] Hara Y., Jahan R.A. (2011). Influence of initial substrate concentration of the Belouzov-Zhabotinsky reaction on transmittance self-oscillation for a nonthermoresponsive polymer chain. Polymers.

[6] Lu X., Zhang H., Fei G., Yu B., Tong X., Xia H., Zhao Y. (2018). Liquid-crystalline dynamic networks doped with gold nanorods showing enhanced photocontrol of actuation. Adv. Mater..

[7] Parrany A.M. (2018). Nonlinear light-induced vibration behavior of liquid crystal elastomer beam. Int. J. Mech. Sci..

[8] Li M.H., Keller P., Li B., Wang X., Brunet M. (2003). Light-driven side-on nematic elastomer actuators. Adv. Mater..

[9] Yu Y., Zhou L., Du C., Zhu F., Dai Y., Ge D., Li K. (2024). Self-galloping of a liquid crystal elastomer catenary cable under a steady temperature field. Thin-Walled Struct..

[10] Zhou L., Chen H., Li K. (2024). Optically-responsive liquid crystal elastomer thin film motors in linear/nonlinear optical fields. Thin-Walled Struct..

[11] Rothemund P., Ainla A., Belding L., Preston D.J., Kurihara S., Suo Z., Whitesides G.M. (2018). A soft, bistable valve for autonomous control of soft actuators. Sci. Robot..

[12] Lv X., Yu M., Wang W., Yu H. (2021). Photothermal pneumatic wheel with high loadbearing capacity. Compos. Commun..

[13] Lendlein A., Jiang H., Jünger O., Langer R. (2005). Light-induced shape-memory polymers. Nature.

[14] Bubnov A., Domenici V., Hamplová V., Kašpar M., Zalar B. (2011). First liquid single crystal elastomer containing lactic acid derivative as chiral co-monomer: Synthesis and properties. Polymer.

[15] Zhou Z., Huang H., Cao D., Qin W., Zhu P., Du W. (2024). Harvest more bridge vibration energy by nonlinear multi-stable piezomagnetoelastic harvester. J. Phys. D Appl. Phys..

[16] Hauser A.W., Sundaram S., Hayward R.C. (2018). Photothermocapillary oscillators. Phys. Rev. Lett..

[17] Kim H., Sundaram S., Kang J.-H., Tanjeem N., Emrick T., Hayward R.C. (2021). Coupled oscillation and spinning of photothermal particles in Marangoni optical traps. Proc. Natl. Acad. Sci. USA.

[18] Chatterjee S. (2011). Self-excited oscillation under nonlinear feedback with time-delay. J. Sound Vib..

[19] Hines L., Petersen K., Lum G.Z., Sitti M. (2017). Soft actuators for small-scale robotics. Adv. Mater..

[20] Sangwan V., Taneja A., Mukherjee S. (2004). Design of a robust self-excited biped walking mechanism. Mech. Mach. Theory.

[21] Qiu Y., Li K. (2024). Self-rotation-eversion of an anisotropic-friction-surface torus. Int. J. Mech. Sci..

[22] Hu W., Lum G.Z., Mastrangeli M., Sitti M. (2018). Small-scale soft-bodied robot with multimodal locomotion. Nature.

[23] Wu H., Ge D., Chen J., Xu P., Li K. (2024). A light-fueled self-rolling unicycle with a liquid crystal elastomer rod engine. Chaos Solitons Fractals.

[24] Kageyama Y., Ikegami T., Satonaga S., Obara K., Sato H., Takeda S. (2020). Light-Driven Flipping of Azobenzene Assemblies—Sparse Crystal Structures and Responsive Behaviour to Polarised Light. Chem.–A Eur. J..

[25] Chun S., Pang C., Cho S.B. (2020). A micropillar-assisted versatile strategy for highly sensitive and efficient triboelectric energy generation under in-plane stimuli. Adv. Mater..

[26] Tang R., Liu Z., Xu D., Liu J., Yu L., Yu H. (2015). Optical pendulum generator based on photomechanical liquid-crystalline actuators. ACS Appl. Mater. Interfaces.

[27] Serak S., Tabiryan N., Vergara R., White T.J., Vaia R.A., Bunning T.J. (2010). Liquid crystalline polymer cantilever oscillators fueled by light. Soft Matter.

[28] Zeng H., Lahikainen M., Liu L., Ahmed Z., Wani O.M., Wang M., Yang H., Priimagi A. (2019). Light-fuelled freestyle self-oscillators. Nat. Commun..

[29] White T.J., Tabiryan N.V., Serak S.V., Hrozhyk U.A., Tondiglia V.P., Koerner H., Vaia R.A., Bunning T.J. (2008). A high frequency photodriven polymer oscillator. Soft Matter.

[30] Akbar F., Rivkin B., Aziz A., Becker C., Karnaushenko D.D., Medina-Sánchez M., Karnaushenko D., Schmidt O.G. (2021). Self-sufficient self-oscillating microsystem driven by low power at low Reynolds numbers. Sci. Adv..

[31] Yang L., Miao J., Li G., Ren H., Zhang T., Guo D., Tang Y., Shang W., Shen Y. (2022). Soft tunable gelatin robot with insect-like claw for grasping, transportation, and delivery. ACS Appl. Polym. Mater..

[32] Wu J., Yao S., Zhang H., Man W., Bai Z., Zhang F., Wang X., Fang D., Zhang Y. (2021). Liquid crystal elastomer metamaterials with giant biaxial thermal shrinkage for enhancing skin regeneration. Adv. Mater..

[33] Boissonade J., De Kepper P. (2011). Multiple types of spatio-temporal oscillations induced by differential diffusion in the Landolt reaction. Phys. Chem. Chem. Phys..

[34] Cicconofri G., Damioli V., Noselli G. (2023). Nonreciprocal oscillations of polyelectrolyte gel filaments subject to a steady and uniform electric field. J. Mech. Phys. Solids.

[35] Kim Y., van den Berg J., Crosby A.J. (2021). Autonomous snapping and jumping polymer gels. Nat. Mater..

[36] Rešetič A., Milavec J., Domenici V., Zupančič B., Bubnov A., Zalar B. (2020). Deuteron NMR investigation on orientational order parameter in polymer dispersed liquid crystal elastomers. Phys. Chem. Chem. Phys..

[37] Charroyer L., Chiello O., Sinou J.-J. (2018). Self-excited vibrations of a non-smooth contact dynamical system with planar friction based on the shooting method. Int. J. Mech. Sci..

[38] Warner M., Terentjev E.M. (2007). Liquid Crystal Elastomers.

[39] Wang Y., Dang A., Zhang Z., Yin R., Gao Y., Feng L., Yang S. (2020). Repeatable and reprogrammable shape morphing from photoresponsive gold nanorod/liquid crystal elastomers. Adv. Mater..

[40] Yang H., Zhang C., Chen B., Wang Z., Xu Y., Xiao R. (2023). Bioinspired design of stimuli-responsive artificial muscles with multiple actuation modes. Smart Mater. Struct..

[41] Bai R., Bhattacharya K. (2020). Photomechanical coupling in photoactive nematic elastomers. J. Mech. Phys. Solids.

[42] Yang L., Chang L., Hu Y., Huang M., Ji Q., Lu P., Liu J., Chen W., Wu Y. (2020). An autonomous soft actuator with light-driven self-sustained wavelike oscillation for phototactic self-locomotion and power generation. Adv. Funct. Mater..

[43] Zhang J., Guo Y., Hu W., Soon R.H., Davidson Z.S., Sitti M. (2021). Liquid crystal elastomer-based magnetic composite films for reconfigurable shape-morphing soft miniature machines. Adv. Mater..

[44] Espíndola-Pérez E.R., Campo J., Sánchez-Somolinos C. (2023). Multimodal and Multistimuli 4D-Printed Magnetic Composite Liquid Crystal Elastomer Actuators. ACS Appl. Mater. Interfaces.

[45] Wang Y., Yin R., Jin L., Liu M., Gao Y., Raney J., Yang S. (2023). 3D-Printed Photoresponsive Liquid Crystal Elastomer Composites for Free-Form Actuation. Adv. Funct. Mater..

[46] Ferrantini C., Pioner J.M., Martella D., Coppini R., Piroddi N., Paoli P., Calamai M., Pavone F.S., Wiersma D.S., Tesi C. (2019). Development of light-responsive liquid crystalline elastomers to assist cardiac contraction. Circ. Res..

[47] Chen B., Liu C., Xu Z., Wang Z., Xiao R. (2024). Modeling the thermo-responsive behaviors of polydomain and monodomain nematic liquid crystal elastomers. Mech. Mater..

[48] Lan R., Shen W., Yao W., Chen J., Chen X., Yang H. (2023). Bioinspired humidity-responsive liquid crystalline materials: From adaptive soft actuators to visualized sensors and detectors. Mater. Horiz..

[49] Agrawal A., Chen H., Kim H., Zhu B., Adetiba O., Miranda A., Cristian Chipara A., Ajayan P.M., Jacot J.G., Verduzco R. (2016). Electromechanically responsive liquid crystal elastomer nanocomposites for active cell culture. ACS Macro Lett..

[50] Liu Y., Wu Y., Liang H., Xu H., Wei Y., Ji Y. (2023). Rewritable Electrically Controllable Liquid Crystal Actuators. Adv. Funct. Mater..

[51] Sun J., Wang Y., Liao W., Yang Z. (2021). Ultrafast, high-contractile electrothermal-driven liquid crystal elastomer fibers towards artificial muscles. Small.

[52] Liao W., Yang Z. (2022). The integration of sensing and actuating based on a simple design fiber actuator towards intelligent soft robots. Adv. Mater. Technol..

[53] Wang Y., Liu J., Yang S. (2022). Multi-functional liquid crystal elastomer composites. Appl. Phys. Rev..

[54] Xu T., Pei D., Yu S., Zhang X., Yi M., Li C. (2021). Design of MXene composites with biomimetic rapid and self-oscillating actuation under ambient circumstances. ACS Appl. Mater. Interfaces.

[55] Manna R.K., Shklyaev O.E., Balazs A.C. (2021). Chemical pumps and flexible sheets spontaneously form self-regulating oscillators in solution. Proc. Natl. Acad. Sci..

[56] Bazir A., Baumann A., Ziebert F., Kulić I.M. (2020). Dynamics of fiberboids. Soft Matter.

[57] Vantomme G., Elands L.C., Gelebart A.H., Meijer E., Pogromsky A.Y., Nijmeijer H., Broer D.J. (2021). Coupled liquid crystalline oscillators in Huygens’ synchrony. Nat. Mater..

[58] Gelebart A.H., Jan Mulder D., Varga M., Konya A., Vantomme G., Meijer E., Selinger R.L., Broer D.J. (2017). Making waves in a photoactive polymer film. Nature.

[59] Shen Q., Trabia S., Stalbaum T., Palmre V., Kim K., Oh I.-K. (2016). A multiple-shape memory polymer-metal composite actuator capable of programmable control, creating complex 3D motion of bending, twisting, and oscillation. Sci. Rep..

[60] He Q., Wang Z., Wang Y., Wang Z., Li C., Annapooranan R., Zeng J., Chen R., Cai S. (2021). Electrospun liquid crystal elastomer microfiber actuator. Sci. Robot..

[61] Xu P., Chen Y., Sun X., Dai Y., Li K. (2024). Light-powered self-sustained chaotic motion of a liquid crystal elastomer-based pendulum. Chaos Solitons Fractals.

[62] Wu H., Dai Y., Li K., Xu P. (2024). Theoretical study of chaotic jumping of liquid crystal elastomer ball under periodic illumination. Nonlinear Dyn..

[63] Baumann A., Sánchez-Ferrer A., Jacomine L., Martinoty P., Le Houerou V., Ziebert F., Kulić I.M. (2018). Motorizing fibres with geometric zero-energy modes. Nat. Mater..

[64] Zhao J., Dai C., Dai Y., Wu J., Li K. (2024). Self-oscillation of cantilevered silicone oil paper sheet system driven by steam. Thin-Walled Struct..

[65] Yu Y., Li L., Liu E., Han X., Wang J., Xie Y.-X., Lu C. (2022). Light-driven core-shell fiber actuator based on carbon nanotubes/liquid crystal elastomer for artificial muscle and phototropic locomotion. Carbon.

[66] Bartlett N.W., Tolley M.T., Overvelde J.T., Weaver J.C., Mosadegh B., Bertoldi K., Whitesides G.M., Wood R.J. (2015). A 3D-printed, functionally graded soft robot powered by combustion. Science.

[67] Wehner M., Truby R.L., Fitzgerald D.J., Mosadegh B., Whitesides G.M., Lewis J.A., Wood R.J. (2016). An integrated design and fabrication strategy for entirely soft, autonomous robots. Nature.

[68] Chen Y., Zhao H., Mao J., Chirarattananon P., Helbling E.F., Hyun N.-s.P., Clarke D.R., Wood R.J. (2019). Controlled flight of a microrobot powered by soft artificial muscles. Nature.

[69] Vantomme G., Gelebart A., Broer D., Meijer E. (2017). A four-blade light-driven plastic mill based on hydrazone liquid-crystal networks. Tetrahedron.

[70] Finkelmann H., Nishikawa E., Pereira G., Warner M. (2001). A new opto-mechanical effect in solids. Phys. Rev. Lett..

[71] Yu Y., Hu H., Dai Y., Li K. (2024). Modeling the light-powered self-rotation of a liquid crystal elastomer fiber-based engine. Phys. Rev. E.

[72] Liu J., Shi F., Song W., Dai Y., Li K. (2024). Modeling of self-oscillating flexible circuits based on liquid crystal elastomers. Int. J. Mech. Sci..

[73] Liu J., Qian G., Dai Y., Yuan Z., Song W., Li K. (2024). Nonlinear dynamics modeling of a light-powered liquid crystal elastomer-based perpetual motion machine. Chaos Solitons Fractals.

[74] Wu H., Zhao C., Dai Y., Li K. (2024). Modeling of a light-fueled self-paddling boat with a liquid crystal elastomer-based motor. Phys. Rev. E.

[75] He Q., Yin R., Hua Y., Jiao W., Mo C., Shu H., Raney J.R. (2023). A modular strategy for distributed, embodied control of electronics-free soft robots. Sci. Adv..

[76] Qiu Y., Chen J., Dai Y., Zhou L., Yu Y., Li K. (2024). Mathematical Modeling of the Displacement of a Light-Fuel Self-Moving Automobile with an On-Board Liquid Crystal Elastomer Propulsion Device. Mathematics.

[77] Zhao D., Liu Y., Liu C. (2017). Transverse vibration of nematic elastomer Timoshenko beams. Phys. Rev. E.

[78] Zhao D., Liu Y. (2019). Effects of director rotation relaxation on viscoelastic wave dispersion in nematic elastomer beams. Math. Mech. Solids.

[79] Jin L., Lin Y., Huo Y. (2011). A large deflection light-induced bending model for liquid crystal elastomers under uniform or non-uniform illumination. Int. J. Solids Struct..

[80] Zhao D., Liu Y. (2019). Photomechanical vibration energy harvesting based on liquid crystal elastomer cantilever. Smart Mater. Struct..

[81] Wei L., Hu J., Wang J., Wu H., Li K. (2024). Theoretical Analysis of Light-Actuated Self-Sliding Mass on a Circular Track Facilitated by a Liquid Crystal Elastomer Fiber. Polymers.

[82] Chartoff R.P., Menczel J.D., Dillman S.H. (2009). Dynamic mechanical analysis (DMA). Thermal Analysis of Polymers: Fundamentals Applications.

[83] Menard K.P., Menard N. (2020). Dynamic Mechanical Analysis.

[84] Lüdde S.C., Dreizler M.R. (2010). Theoretical Mechanics.

[85] Truesdell C. (2012). Essays in the History of Mechanics.

[86] Dugas R. (1988). A History of Mechanics.

[87] Yu Y., Nakano M., Ikeda T. (2003). Directed bending of a polymer film by light. Nature.

[88] Herbert K.M., Fowler H.E., McCracken J.M., Schlafmann K.R., Koch J.A., White T.J. (2022). Synthesis and alignment of liquid crystalline elastomers. Nat. Rev. Mater..

[89] Nägele T., Hoche R., Zinth W., Wachtveitl J. (1997). Femtosecond photoisomerization of cis-azobenzene. Chem. Phys. Lett..

[90] Torras N., Zinoviev K., Marshall J., Terentjev E., Esteve J. (2011). Bending kinetics of a photo-actuating nematic elastomer cantilever. Appl. Phys. Lett..

[91] Zhao T., Zhang Y., Fan Y., Wang J., Jiang H., Lv J.-A. (2022). Light-modulated liquid crystal elastomer actuator with multimodal shape morphing and multifunction. J. Mater. Chem. C.

